# Congenital hemivertebrae combined with situs inversus totalis: A rare case report

**DOI:** 10.1097/MD.0000000000037625

**Published:** 2024-03-29

**Authors:** Zheng Guo, Donglai Li, Xuehui Zang

**Affiliations:** a Orthopedics Department of the Sixth Affiliated Hospital, School of Medicine, South China University of Technology, Foshan, China; b Department of Orthopaedics, Qilu Hospital of Shandong University, Jinan, People’s Republic of China.

**Keywords:** case report, congenital hemivertebrae, situs inversus totalis, spine

## Abstract

**Rationale:**

Situs inversus totalis is a rare malposition of organs that typically involves lesions in the respiratory, circulatory, or urinary systems. Cases of congenital hemivertebrae combined with situs inversus totalis are extremely rare and have limited reports.

**Patient concerns:**

We report a 2.5 years old girl with 2 congenital hemipyramids and complete visceral inversion who ultimately underwent hemilaminectomy.

**Diagnosis:**

Congenital hemivertebrae combined with situs inversus totalis.

**Intervention:**

The patient underwent hemilaminectomy.

**Outcomes:**

The spinal deformity was corrected.

**Lessons:**

For patient with spinal deformities combined with situs inversus totalis, surgery can be an effective treatment method. But we also need to be vigilant about the dysfunction of various systems.

## 1. Introduction

Situs inversus totalis is a rare malposition of organs in the chest and abdomen with a prevalence of 1/5000–1/10,000 among population.^[1]^ It is characterized by the reversal of the organs in the mirror image of the normal arrangement.^[2]^ Situs inversus totalis reported in current literature is often associated with congenital heart disease,^[3]^ kidney malformation,^[1]^ umbilical cord dysplasia,^[4]^ chronic sinusitis/nasal polyps, and bronchiectasis (Kartagener syndrome, KS).^[5]^ Some researchers suggested that the physical and chemical factors and mutant genes leading to situs inversus totalis may be also the reason for the tethered spinal cord, split cord malformation and Klippel Feil syndrome.^[6]^ However, there are few reports of situs inversus totalis combined with scoliosis worldwide.^[5]^ As such, we present a 2.5 years old girl with 2 congenital hemivertebrae and situs inversus totalis who underwent hemilaminectomy in our hospital.

## 2. Case presentation

**Figure 1.**
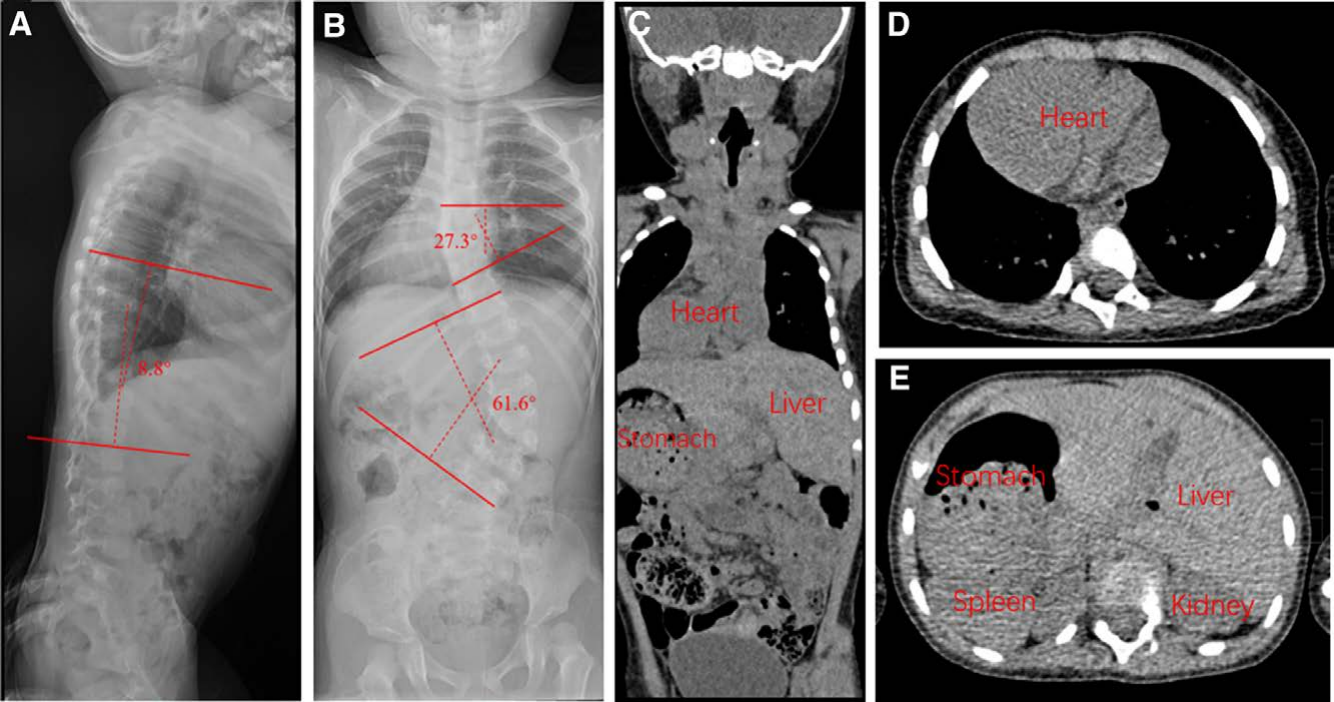
Radiographic features of the patient. (A) Lateral full spine X-ray and TK. (B) Standing full spine X-ray and Cobb angle of thoracic and lumbar curve. (C) Sagittal plane of CT. (D and E) CT of chest and abdomen. CT = computed tomography, TK = thoracic kyphosis.

A 2.5 years old girl was admitted to our hospital for her spinal deformity and surgeons were informed of the girl’s situs inversus totalis. The child’s growth and nutritional status were as normal as her peers and no neurological symptoms were observed. The apex cardiac beat was on the right and no coffee spots or abnormal hair growth showed on her body. The left hand had 6 fingers and the extra finger was attached to the metacarpophalangeal joint on her left hand. Hemivertebrae between T6–7 and L2–3 were observed on the X-ray film. The Cobb angle of the thoracic curve was 27.3° and lumbar curve was 61.6°, and thoracic kyphosis was 8.8° (Fig. 1). Computed tomography showed a right-side heart with an atrial septal defect and gastric and spleen were on the right side, while the liver was on the left side (Fig. 1). The echocardiogram also suggested a right-sided heart with an atrial septal defect and 60% of ejection fraction. No significant abnormalities were found in her hematology examination, abdominal, and genitourinary ultrasound examination.

The patient was diagnosed with congenital hemivertebrae, situs inversus totalis, atrial septal defect, and polydactyly. Before the operation, the patient was evaluated by cardiologists and hand surgeons for her atrial septal defect and polydactyly. And no specific treatments were required for her atrial septal defect. Due to vast estimated intraoperative bleeding and long surgery time, the extra finger will not be removed until complete recovery from the current surgery.

The lumbar hemivertebra was resected and the adjacent segments were fixed. Two pedicle screws were implanted on the adjacent vertebrae respectively, and then the hemivertebrae body was resected using eggshell procedure. The titanium rods and towers were placed, and the gap was closed by applying pressure to screws, then lock the system. The bone of vertebrae was not so solid that the screw moved slightly during pressurization. A brace was dressed immediately after operation. The postoperative lumbar Cobb angle was 8°, and the correction rate was 81.4%.

## 3. Discussion

Situs anomalies include situs ambiguous and situs inversus totalis. The former presents as disarrangement of the internal organs across the left-right axis of the body; whereas the latter’s organs keep the normal relationship with each other.^[1]^ The computed tomography of this patient we reported showed the heart apex, gastric, and spleen on the right side, while the liver was on the other side, suggesting the diagnosis is situs inversus totalis. Patients with situs inversus totalis often search for medical service for KS, congenital heart disease, and urinary system disorders, but it is rare of combining with hemivertebrae or scoliosis (Table 1).

**Table 1:** Dextrocardia/situs inversus totalis with spinal dysraphism in literature and our case.

Author, yr	Age	Gender	Scoliosis type	Complications
Davies et al, 1965^[7]^	32	Female	Hemivertebrae	Dextrocardia, rib deformities, mitral stenosis
Tubbs et al, 2003	11	Female	Hemivertebrae	Situs inversus totalis, Spina bifida occulta, type II split cord malformation, sacral lumbarization
Nallegowda et al, 2003^[8]^	12	Male	Congenital scoliosis	Situs inversus totalis, bilateral radial club hands, hypospadias, isolated dextrocardia, hypoplastic ribs, ectopic kidney, spina bifida occulta
Gul et al, 2006^[4]^	Fetus	Male	Hemivertebrae	Dextrocardia, polydactyl, atrial septal defect, hypoplastic ribs
Yu et al, 2009^[5]^	11	Male	Congenital scoliosis	Situs inversus totalis, type II split cord malformation, spina bifida occulta, hypoplastic ribs, coffee spots
Yu et al, 2009^[5]^	12	Female	Hemivertebrae	Situs inversus totalis, spina bifida occulta, coffee spots, abnormal hairs
Yu et al, 2009^[5]^	3	Male	Hemivertebrae	Situs inversus totalis, spina bifida occulta
María et al, 2014	14	Male	Unknown	Situs inversus totalis
Zhu et al, 2017^[1]^	9	Female	Hemivertebrae	Situs inversus totalis, syringomyelia, congenital heart defect, pulmonary dysfunction, hydronephrosis
Alkin et al, 2018	22	Female	Unknown	KS
Guo et al, 2022^[9]^	19	Male	Idiopathic scoliosis	KS, smell and hearing defects, Asthenoteratozoospermia
Guo et al, 2022^[9]^	37	Female	Idiopathic scoliosis	KS, smell and hearing defects, immature uterus
Our case, 2022	2	Female	Hemivertebrae	Situs inversus totalis, atrial septal defect, polydactyl

KS = Kartagener syndrome.

Mesenchymal vertebrae will transform to chondrification centers during the 6th week of fetal growth and then, vertebrae develop.^[4]^ If the vertebrae formation failed, hemivertebrae or butterfly vertebrae would appear as a consequence, which could result in scoliosis.^[10]^ Obvious hemivertebrae can be detected by prenatal ultrasound screening. However, spinal deformity or hemivertebrae do not come to attention in early infancy until late infancy or childhood period, when scoliosis gradually worsens and spine deformity becomes more serious. Generally, patients were first diagnosed at the mean age of 1.9 years,^[4]^ while the girl’s scoliosis in this case was noticed at the age of 1 year and 7 months when she came to hospital for her cough. However, the respiratory symptoms were transient, so KS was excluded.

The mechanism of scoliosis combined with situs inversus totalis remains unclear. Kouwenhoven et al,^[11]^ studied the lumbar magnetic resonance imaging of 37 patients with scoliosis and situs inversus totalis, founding that their mid and lower thoracic vertebrae (T5–12) mildly rotated to the left. The majority of them were right-handed, suggesting that the vertebral rotation is related to visceral position rather than hand use habits. Generally, patients with idiopathic scoliosis exhibit right thoracic curve and vertebrae’s rightward rotation; however, in 2 studies of 4 patients with idiopathic scoliosis and situs inversus by Guo et al^[9]^ and Lu et al,^[12]^ the thoracic curve bent to the left and the vertebrae of mid and lower thoracic vertebrae rotated to the left in patients with situs inversus totalis, which supported that the curve direction and vertebrae rotation were mainly determined by organs distribution.

Previous studies have found that systemic abnormalities may originate from a specific common mutation gene. Schlösser et al^[13]^ reported the prevalence of scoliosis in patients with situs inversus totalis reached 8%, which is significantly higher than the normal population (1.5–3%), indicating that the 2 diseases may share a common physicochemical or molecular biological basis. Tubbs et al^[14]^ presented a case of split cord malformation combined with situs inversus totalis and reviewed the relevant literature and found that multiple deformities were usually observed in this group of patients. Therefore, they made a hypothesis that multiple abnormalities may share a common initial factor. Yu et al^[5]^ reported 3 patients suffered scoliosis and situs inversus totalis and drew a conclusion that the deformity at the mid-axis may be correlated with situs inversus totalis, so they pointed out that congenital scoliosis and situs inversus totalis or right-sided heart may be related to genes involved in determining the development of the mid-axis site.

Ting Guo et al^[9]^ and Lu et al^[12]^ performed whole exome capture and high-throughput sequencing in 4 patients diagnosed with primary ciliary dyskinesia, who presented with KS and scoliosis (Cobb angle > 10°) and found disease-causing variants in *DNAAF2* and *DNAAF4. DNAAF2* and *DNAAF4* are genes of the kinesin assembly subunits, which are essential for ciliary motility. Meanwhile, some studies indicated that sensory-related structures, including motor cilia, neurons in contact with cerebrospinal fluid, Reissner fibers and Urotensin neuropeptide signaling, had a potential impact on the development of the perceptual axis^[15]^; and it has also been suggested that some gene mutations were related with abnormal ciliary beat and scoliosis. Therefore, we speculate that cilia-related genes may be the common molecular biological basis for scoliosis and situs inversus totalis. However, the concrete gene mutation locations and the mechanism remain unclear, thus future genetic and developmental studies are needed to reveal the pathogenesis.

It is not difficult to diagnose situs inversus totalis clinically. However, patients with situs inversus totalis should be carefully examined to detect potential abnormalities. Operation procedure or method of the posterior spine or symmetrical organs will not be influenced by situs inversus totalis, but the surgical approach should be carefully evaluated when performing anterior spine surgery or asymmetrical organ surgery.

## Author contributions

**Data curation:** Zheng Guo, Donglai Li.

**Methodology:** Xuehui Zang.

**Resources:** Zheng Guo, Donglai Li.

**Supervision:** Xuehui Zang.

**Visualization:** Xuehui Zang.

**Writing – original draft:** Zheng Guo.

**Writing – review & editing:** Xuehui Zang.
